# Data-driven design and controllable synthesis of Pt/carbon electrocatalysts for H_2_ evolution

**DOI:** 10.1016/j.isci.2021.103430

**Published:** 2021-11-13

**Authors:** Anhui Zheng, Yuxuan Wang, Fangfei Zhang, Chunnian He, Shan Zhu, Naiqin Zhao

**Affiliations:** 1 School of Materials Science and Engineering, Tianjin University, Tianjin 300350, China; 2 Joint School of National University of Singapore and Tianjin University, International Campus of Tianjin University, Binhai New City, Fuzhou 350207, China

**Keywords:** Chemical reaction, Catalysis, Materials science, Computational method in materials science

## Abstract

To achieve net-zero emissions, a particular interest has been raised in the electrochemical evolution of H_2_ by using catalysts. Considering the complexity of designing catalyst, we demonstrate a data-driven strategy to develop optimized catalysts for H_2_ evolution. This work starts by collecting data of Pt/carbon catalysts, and applying machine learning to reveal the importance of ranking various features. The algorithms reveal that the Pt content and Pt size have the greatest impact on the catalyst overpotentials. Following the data-driven analysis, a space-confined method is used to fabricate the size-controllable Pt nanoclusters that anchor on nitrogen-doped (N-doped) mesoporous carbon nanosheet network. The obtained catalysts use less platinum and exhibit better catalytic activity than current commercial catalysts in alkaline electrolytes. Moreover, the data formed in this work can be used as feedback to further improve the data-driven model, thereby accelerating the development of high-performance catalysts.

## Highlights

• Built a database of Pt/C catalysts for H_2_ evolution
• Provided quantitative guidance for catalyst design by machine learning
• Developed a space-confined strategy to control the features of Pt/C composites
• Formed a closed-loop from data-driven design to catalyst evaluation

## Graphical abstract

**Figure:**
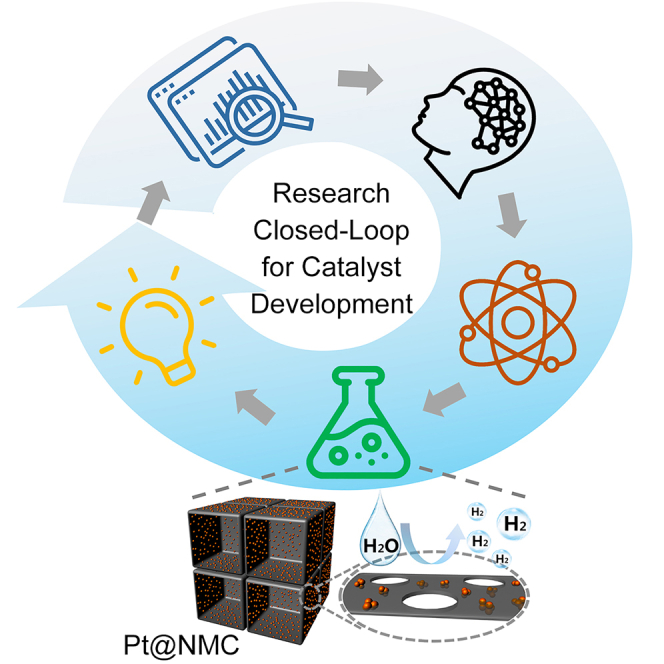

## Introduction

To meet the growing energy demand and achieve the net-zero emissions, hydrogen energy has received widespread attention due to the high calorific value and pollution-free characteristics of H_2_ (Kibsgaard and Chorkendorff, 2019; Staffell et al., 2019). Especially, using the electricity generated by solar and wind power to electrolyze water paves the way for the large-scale application of H_2_ (Buttler and Spliethoff, 2018; You and Sun, 2018). The H_2_ evolution reaction (HER) is a key step in the water splitting, whose efficiency and energy consumption are restricted by the performance of the catalyst. Therefore, the development of highly active, stable and low-cost catalysts is essential to promote the hydrogen energy applications (Seh et al., 2017; Zhu et al., 2020).

Currently, the most used HER catalysts are Pt-based materials, and many factors have been involved in their optimization (Xu et al., 2018). Owing to the scarcity and the high price of Pt, its usage should be reduced as much as possible under the premise of ensuring the catalytic effect (Li et al., 2019). To balance the catalytic activity and the cost, researchers used to reduce the metal size to nanometer to achieve the maximum efficiency of atoms (Zhang et al., 2021). However, the nanosized catalysts need to be supported on a support such as the carbon material to maintain the reaction stability and improve the charge/mass transfer (Yang et al., 2020; You and Sun, 2018). Overall, all these factors, for example, Pt content, Pt size, the physical, and chemical properties of the carbon support, impact the actual performance of HER catalysts (Liang et al., 2019). Not to mention the current density and the selected electrolyte in the actual tests (Liang et al., 2020; Zheng et al., 2016). Faced with numerous influencing factors, different researchers have their own focuses, such as enhancing the intrinsic catalytic activity by controlling the crystalline or atomic state of Pt, or improving the reaction efficiency by constructing elaborate carbon support nanostructures (Wan et al., 2020; Lai et al., 2016; Suliman et al., 2019). These studies do drive the improvement of catalysts, yet only a limited number of features can be observed in the separate experiments. In addition, there is a lack of continuous improvement of the catalyst-design process. Therefore, it is challenging to construct a research framework that can achieve a more comprehensive analysis of the catalyst system and enable a continuous evolutionary upgrade.

For the complex system of catalyst, a promising problem-solving strategy is to change the research paradigm from experience/theory-driven to data-driven (Butler et al., 2018; Li et al., 2020; Tran and Ulissi, 2018; Zhu et al., 2021). Especially, the data-driven machine learning (ML) methods are rising in the field of catalyst design (Lin et al., 2020; Sun et al., 2020; Wu et al., 2020). The typical ML-based research is to analyze the input data (the features of materials) and the output data (the research targets) by the algorithms, and their intrinsic relationship can be obtained without the need to fully understand the physical and chemical mechanisms (Li et al., 2020; Lu et al., 2020). The difficulty of this strategy lies in how to obtain the appropriate data and how to select the suitable evaluation descriptor (Batra, 2021; Toyao et al., 2020). For the input, the characteristics of the support and the test conditions are missing in most cases. The supplement of this part of information can enhance the ability of ML to analyze the HER catalyst systems. For the output, most of the existing studies use descriptors such as hydrogen adsorption energy as the label/target. These types of descriptors can indeed reflect the intrinsic properties of materials, yet they do not have the simple linear relationship with the catalyst-measured performance. If the performance criteria, for example, overpotential (η), are directly used as the output target, the application potential of the catalysts can be evaluated more efficiently and accurately.

Another challenge of data-driven catalyst development is how to obtain the target materials designed by the algorithms. To this regard, we need a universal synthesis approach to achieve the control of Pt particle size, loading amount, and other parameters. At the same time, the properties of the carbon support need to be considered. At least, the carbon support should form a stable anchoring state with Pt nanoparticles, ensuring the stability of the H_2_ evolution (Tavakkoli et al., 2017). In addition, the architecture of the carbon matrix should be adept with the gas-solid-liquid interfaces when HER happens (Zhu et al., 2019). Moreover, the parameters such as the microstructure and surface state of the support should be controllable in order to follow the data-driven models.

Integrating intelligent analysis and controllable synthesis, we propose a data-driven research framework to develop the high-performance HER catalysts. In this work, we collect data from the reported researches and apply ML algorithms to design the catalysts that are suitable for catalyzing HER. According to the algorithm analysis, an N-doped mesoporous carbon nanosheet network (NMC) is fabricated by the salt template method and used as the support to realize the controllable preparation of Pt nanoclusters (∼1 nm). The electrochemical tests show that the catalytic performance of the obtained composite (Pt@NMC) in alkaline electrolyte is superior to that of the commercial Pt/C. Moreover, the new data from this work can be used as a supplement to the original database, thus forming a closed-loop framework to accelerate the discovery of HER catalysts.

## Results and discussion

### Data mining and machine learning

**Figure 1:**
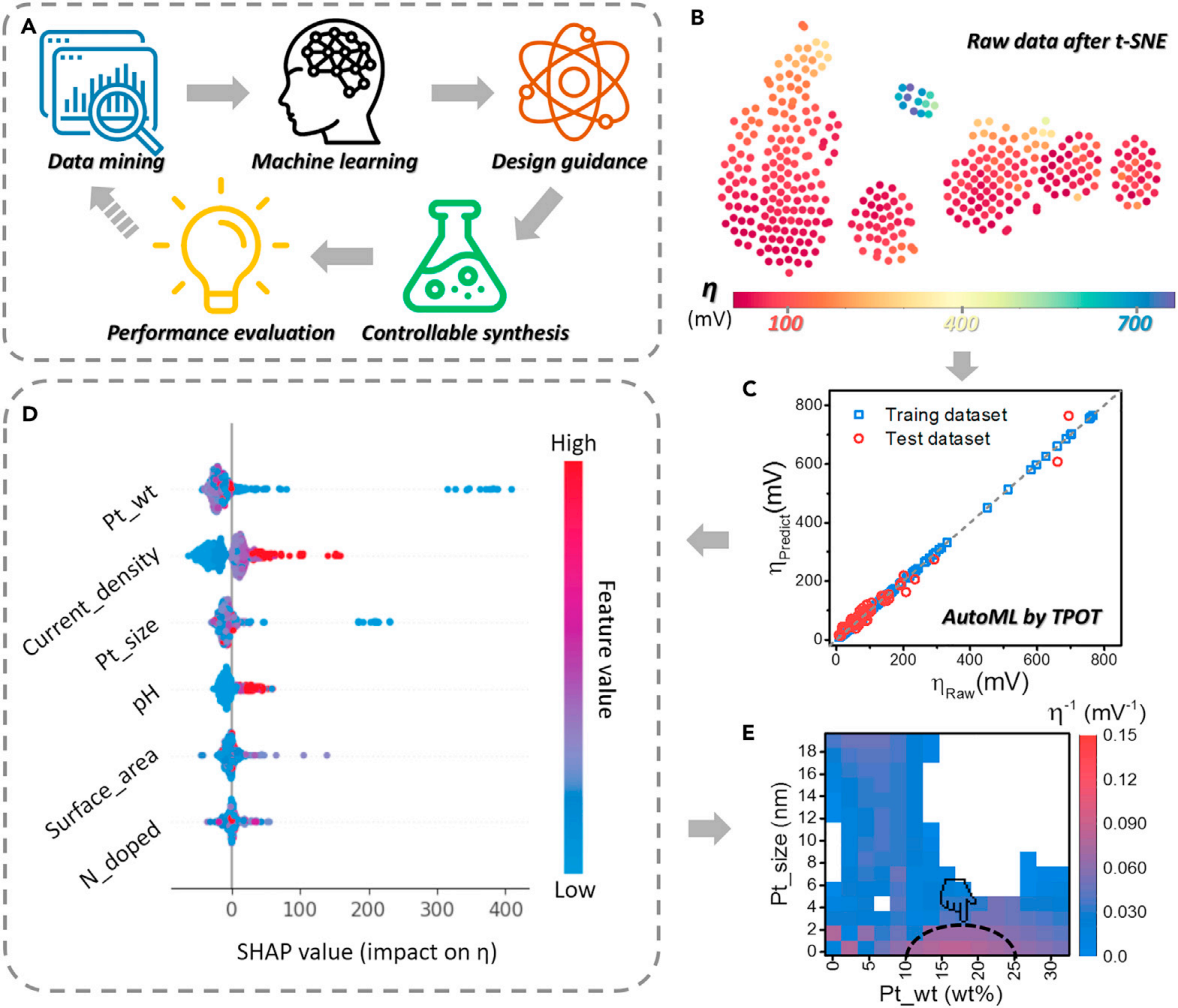
The research pipeline and the machine learning process (A) Schematic diagram of data-driven H_2_ evolution catalyst development research pipeline. (B) The collected data was conducted dimensionality reduction by t-SNE. (C) Machine learning results processed by TPOT algorithm. (D) The Shapley value (SHAP) of each feature in optimization algorithm. (E) Heatmap showed the influence of Pt_wt and Pt_size on overpotential: the overpotential was in the form of reciprocal (η^−1^), and the dotted box pointed by the finger was the zone of best performing samples.

Firstly, we established a research pipeline from initial data mining to final catalyst performance evaluation (Figure 1A). The start of this pipeline is the data mining/collection. In this step, we extracted the test results from real experiments covering as many influencing factors as possible. As the result, more than 200 sets of Pt/C catalysts for HER were collected from hundreds of published papers. For each sample, the features include the characters of Pt (*e.g.* content and particle size), the details of carbon support (*e.g.* specific surface area, N-doped level, I_D_/I_G_, and pore volume), the information of test system (*e.g.* electrolyte, test current density, and mass loading), and the corresponding catalytic performance (*e.g.* overpotential, mass activity, turnover frequency). Also, it needs to be mentioned that the data collection cannot cover all influence factors, which leaves room for future completion. All these data were available in the supplemental information (Data S1. Database of Pt/C catalysts, Related to STAR Methods).

With the database derived from the real experiments, the features were screened according to the existed scientific understanding. For the part of Pt, two main features were remained, that is, Pt content (Pt_wt) and Pt particle size (Pt_size). Because Pt is the active component that directly catalyzes HER, its content is one of the most important factors (Kibsgaard and Chorkendorff, 2019; Buttler and Spliethoff, 2018). Meanwhile, the particle size of Pt greatly impacts the number of active sites and the catalytic stability (Seh et al., 2017; Xu et al., 2018). Considering the interaction between Pt and its carbon support, two features relating to the carbon matrix were picked up in this research, that is, the specific surface area (surface_area) and N-doped content (N_doped). During the catalytic reaction, these factors affect the active site distribution, the catalytic center, and the charge/mass transfer processes (Yang et al., 2020; Zhu et al., 2019). Additionally, the electrochemical testing conditions should be taken into consideration, especially the pH of electrolyte (pH) and the externally applied current (current_density). The former influences the reaction mechanism on the catalyst surface in HER, while the latter affects the consumption of internal resistance and the catalytic efficiency (Xu et al., 2018). There is no further processing on these features, such as principal component analysis, because of two reasons: (1) the number of selected features is small, a stricter screening will reduce the universality; (2) it can improve the interpretability of the model in the consequent analysis. For the output target, there are several candidates, such as overpotential, Tafel slope, and charge transfer resistance (R_ct_) from EIS data (Figure S1). In general, Tafel slope represents the kinetic process of catalysis; R_ct_ indicates the charge transfer ability of the system. Comparing with these indicators, the overpotential in the actual test directly reflects the catalyst performance, and is chosen as the output target in this study (Kibsgaard and Chorkendorff, 2019; Staffell et al., 2019). These data of ML were summarized in the supplemental information (Data S2. Dataset of Pt/C catalysts used in ML models, Related to Figure 1), whose dimensions were reduced by the t-SNE algorithm for the visualization (Figure 1B).

Given the input features and the output target, we can find that the relationships between the factors and the overpotential are non-linear (Figure S2). For this complex system, the Tree-based Pipeline Optimization Tool (TPOT) was applied for the following analysis. TPOT is a kind of automated ML (AutoML) tool that allows researchers to automatically perform repetitive steps in ML cases (Olson et al., 2016). More importantly, TPOT can create a benchmark to act on the complex tasks in a standardized manner (Chen et al., 2020). We divided the database to 80% training set and 20% test set, and run TPOT to explore thousands of possible models and find the best one for our data (the details were described in the experimental section). After the TPOT optimization, the selected algorithm was Gradient Boosting Regressor, and the specific parameters were listed in Table S1. Using the optimized ML model, the high prediction accuracy can be achieved: the r^2^ score is 0.99 for the training set and 0.97 for the test set (Figure 1C). Meanwhile, the results of mean squared error (MSE) and the mean absolute error (MAE) were listed in Table S2. Because we have included the overpotential under various current densities in the database, the numerical value of the overpotential fluctuates largely; thus, the MSE and MAE are relatively high. In addition, the score and the relative errors for training and test dataset during the training progress were plotted in Figure S3. To further verify the reliability of the algorithm, we carried out K-fold cross-validation, whose results prove that the division of datasets does not affect the regression score of the selected ML model (Table S3).

Owing to their “black box” characteristics, the ML-based researches often face the problem of explanatory (Butler et al., 2018; Li et al., 2020). Herein, we adopted a method derived from the game theory, namely SHAP (Shapley additive explanations), to explain the obtained ML results (Lundberg and Lee, 2017). When analyzing a multi-feature system, the Shapley value can be used to calculate the contribution of each feature to the final output (Lundberg et al., 2020). The SHAP analysis of this study was plotted in a honeycomb diagram (Figure 1D). In general, the SHAP values of the selected features present three types of distribution. Regarding the catalyst itself (Pt_wt and Pt_size), their influence patterns on the output of η are complicated. In the series of Pt_wt, for samples with high feature values, their Shapley values tend to be in the low-value area, which means that high Pt content corresponds to low overpotential and high catalytic activity (Figure S4A). This phenomenon fits our general intuition. However, in several cases (such as the catalyst using single Pt atoms), the samples with low loading of Pt exhibit high activity. These seemingly contradictory data once again prove the complexity of the catalyst system. For the test conditions, the distinction of their SHAP values distribution is significant, meaning that these two factors (pH and current_density) had the clear influence on the catalytic activity. Taking the pH as an example, in general trend, the higher pH corresponds to the higher SHAP values, suggesting that the alkaline environment tended to increase the overpotential (Figure S4B). Considering that the most used electrolytes in the current industrial hydrogen production are alkaline, improving catalyst activity in high pH environments is a meaningful challenge (Holladay et al., 2009). Compared with the aforementioned features, the SHAP values relative to the carbon support (surface_area and N_doped) are more concentrated in the central area, suggesting that their contribution to catalytic activity is relatively low. We calculated the absolute value of each SHAP value and counted their average to obtain the impact of each feature (Figure S5). The larger the average value, the higher the weight of the feature influence on the output. Obviously, Pt_wt and Pt_size share the most prominent factors related to the active material. In addition, the weight of current density is significant, whose impact is higher than that of the electrolyte properties.

To provide guidance for consequent synthesis, we extracted the most critical features and combined them with the overpotential to form a heatmap (Figure 1E). To make the results more intuitive, the reciprocal of overpotential (η^−1^) was used as the basis for plotting: the higher the η^−1^, the better the catalyst activity of Pt/C. When generating the heatmap, the algorithm will homogenize the values of adjacent regions and cause some regions presenting the values less than 0. Because the η^−1^ less than 0 have no physical meaning, we removed them of the display and plotted Figure 1E (the heatmap with automatically default range was shown in Figure S6). As the results, the range of the “better performing” Pt content is between 10 wt % and 25 wt %; for the feature of Pt_size, the particle sizes of below 2 nm have the greatest potential. Similarly, for the carbon support, the heatmap was constructed by surface_area, N_doped, and η^−1^. Because the distributions of N_doped and surface_area in the database are concentrated, only some local optimization areas are founded, such as 500–1200 m^2^ g^−1^ for surface_area and 5–12 wt % for N_doped (Figure S7).

### Controllable synthesis

**Figure 2:**
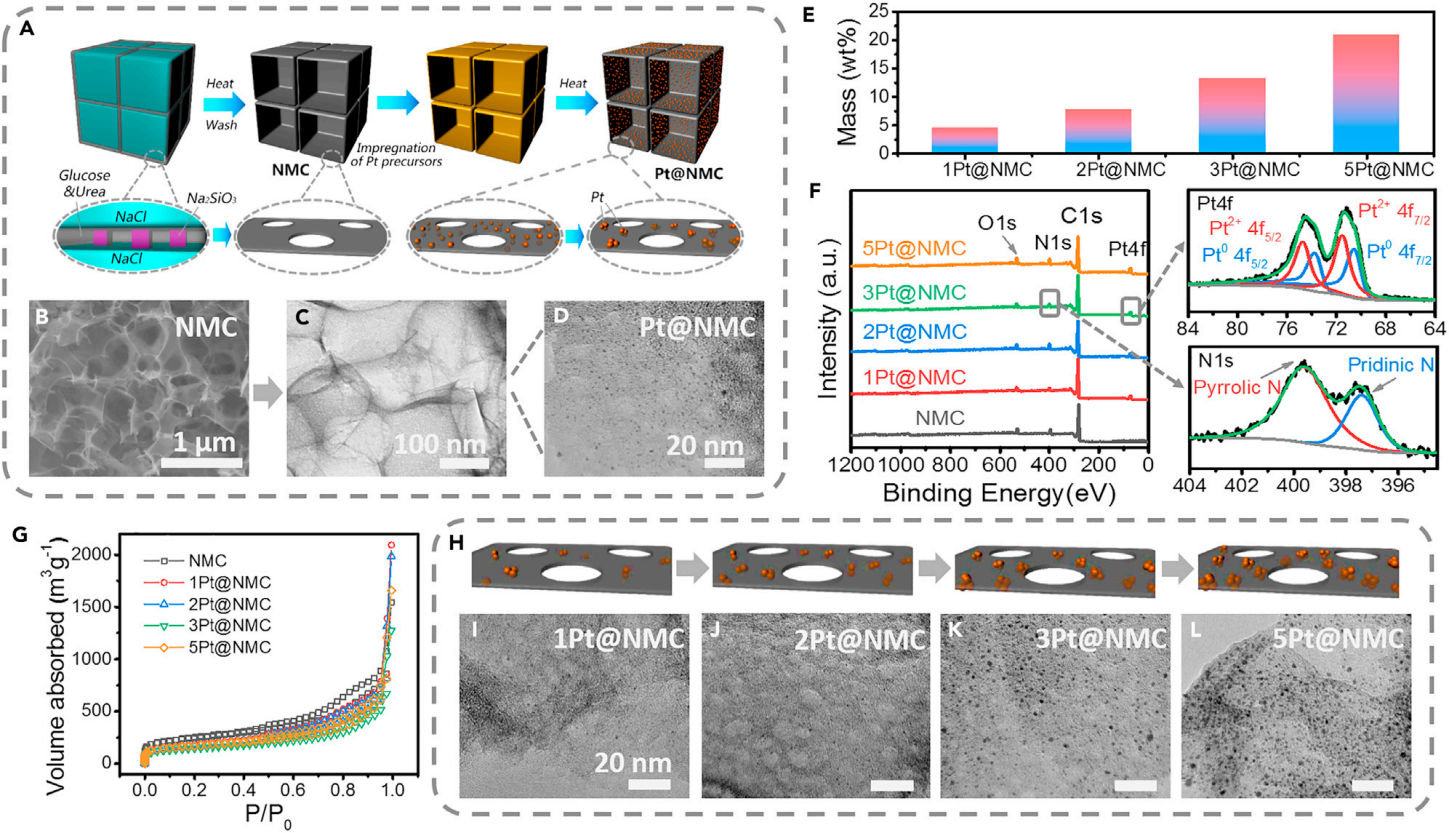
The synthesis of Pt@NMC catalysts (A) Schematic diagram of controllable synthesizing Pt@NMC catalysts. (B) SEM image of NMC (the scale bar is in the figure). (C) TEM image of Pt@NMC (the scale bar is in the figure). (D) TEM image of Pt nanoclusters and mesopores in Pt@NMC (the scale bar is in the figure). (E) TGA results of Pt@NMC samples. (F) XPS results of Pt@NMC samples. (G) N_2_ adsorption-desorption isotherm of NMC and the series of Pt@NMC. (H–L) (H) Schematic diagram of Pt nanoclusters evolution in Pt@NMC and (I-L) corresponding TEM images (the scale bar is in the figure).

On the basis of ML results, we aimed to prepare samples that fit the intelligent guidelines. To this regard, a carbon space-confined strategy was developed to fabricate content-controllable and size-controllable nano-Pt (Figure 2A). Firstly, a nitrogen-doped mesoporous carbon nanosheet network (NMC) was produced by using water-soluble salts as the templates (Zhu et al., 2015). The obtained NMC has two types of pores: (1) the macropores (1–2 μm) derived from NaCl contribute to its 3D network architectures; (2) the mesopores (5–10 nm) caused by NaSi_2_O_3_ are evenly distributed on the wall of the macropores (Figures 2B and S8). Both TEM observation and the two-dimensional (2D) peaks in Raman spectrum (Figure S9) prove the ultrathin characteristics of the carbon nanosheets. Next, the obtained NMC adsorbed Pt ion precursors by an immersion step, and these ions were reduced by following high-temperature annealing. During this process, Pt atoms went through a limited agglomeration process in the ultrathin carbon nanosheets, and converted to nanoclusters without damaging the original structure of NMC (Figure 2C). Moreover, STEM indicates (Figure S10) that Pt elements are evenly distributed in the carbon matrix, and HRTEM (Figures 2D and S11) reveals that the Pt nanoclusters with controllable size (∼1 nm) are anchored on NMC to obtain the target composite (noted as Pt@NMC).

We further characterized the structure and composition of the Pt@NMC samples. X-ray diffraction peaks verify the removal of salt and the successful introduction of Pt (Figure S12). TGA results (Figures 2E and S13) show that the increase of the Pt precursor concentration (0.01M, 0.02M, and 0.03M) during the immersion results in the increased Pt content (4.6 wt %, 7.7 wt %, and 13.3 wt %) in the composites. When the concentration reached 0.05M (5Pt@NMC), the loaded Pt content is 21 wt % that is close to the value of commercial Pt/C catalyst (20 wt %). All these prove that the Pt content is controlled within the optimal range by the NMC space-confined strategy. Besides, X-ray photoelectron spectrum (XPS) (Figure 2F) demonstrates that the co-existence of Pt^0^ and Pt^2+^ in the metal nanoclusters leads to higher intrinsic catalytic activity than common Pt nanoparticles (Wan et al., 2020). Meanwhile, the N-doped contents in the carbon matrix, introduced by the decomposition of urea, are between 6.3 wt % and 10.4 wt %. This heteroatoms doping has two benefits: (1) forming a stable complex structure with Pt ions during immersion step (He et al., 2013; Ying et al., 2017); (2) increasing the wettability of Pt@NMC with aqueous electrolytes (Wang and Xiao, 2018). Meanwhile, the ratio of D peak (∼1350 cm^−1^) to G peak (∼1580 cm^−1^) in Raman results, noted as I_D_/I_G_, can be used to characterize the crystallinity of carbon matrix (Zhu et al., 2015). The I_D_/I_G_ of NMC is calculated to 0.98, and these values of the Pt@NMC samples are stable in the range of 0.91–0.99 (Table S4), indicating that the introduction of Pt does not significantly affect the crystallinity of the carbon matrix. Furthermore, the high specific surface area of NMC (864 m^2^ g^−1^) is kept in Pt@NMC (ranging from 516 to 641 m^2^ g^−1^) (Figure 2G and Table S5). The pore size distribution, consistent with the TEM characterization, shows the existence of mesopores (∼10 nm) in Pt@NMC (Figure S14), which are derived from the Na_2_SiO_3_ template during fabricating NMC (Zhu et al., 2015). Thanks to these high surface areas and abundant mesopores, the open architecture of Pt@NMC is beneficial to not only the diffusion of electrolyte ions but also the rapid escape of gas (Lai et al., 2016; Suliman et al., 2019).

To understand the space-confined effect of NMC in synthesis, we studied the morphological evolution of Pt@NMC in various precursor concentrations (Figures 2H–2L and S15). The nanosized Pt formation can be summarized as that the Pt ions in NMC are reduced and agglomerate into nanoclusters at high temperatures. During this process, the limited space constructed by the unique structure of NMC should be taken into account. Thus, the Pt agglomeration is confined by two aspects: (1) the 2D properties of the carbon nanosheets hinder the motion of Pt on the vertical scale (Zhu et al., 2017); (2) the mesopores in NMC block the long-range horizontal movement of Pt atoms. Combining these two space-confined effects, the final size of Pt is effectively regulated at about 1 nm (from 0.88 to 1.07nm), even though the Pt content is elevated by adding the precursor concentration (Figures 2I–2L and S16).

In addition to Pt content and Pt particle size, our method can also control other key parameters. For example, the N-doped level can be adjusted by changing the relative content of urea. In the preparation of the control sample, the mass ratio of glucose to urea is changed to 1:1, 1:0.5, and 1:0.1, the N-doped content of the corresponding product is 10.4 wt%, 8.2 wt%, and 5.8 wt% (Figure S17). The last two samples were named as Control-1 and Control-2, respectively. Their microstructures, N-doped contents, and Pt loading amount were summarized in Table S6. Indeed, there is not a simple linear relationship between the raw materials and the composition of products, and the specific fine-tune needs follow-up research.

### Catalyst performance evaluation

**Figure 3:**
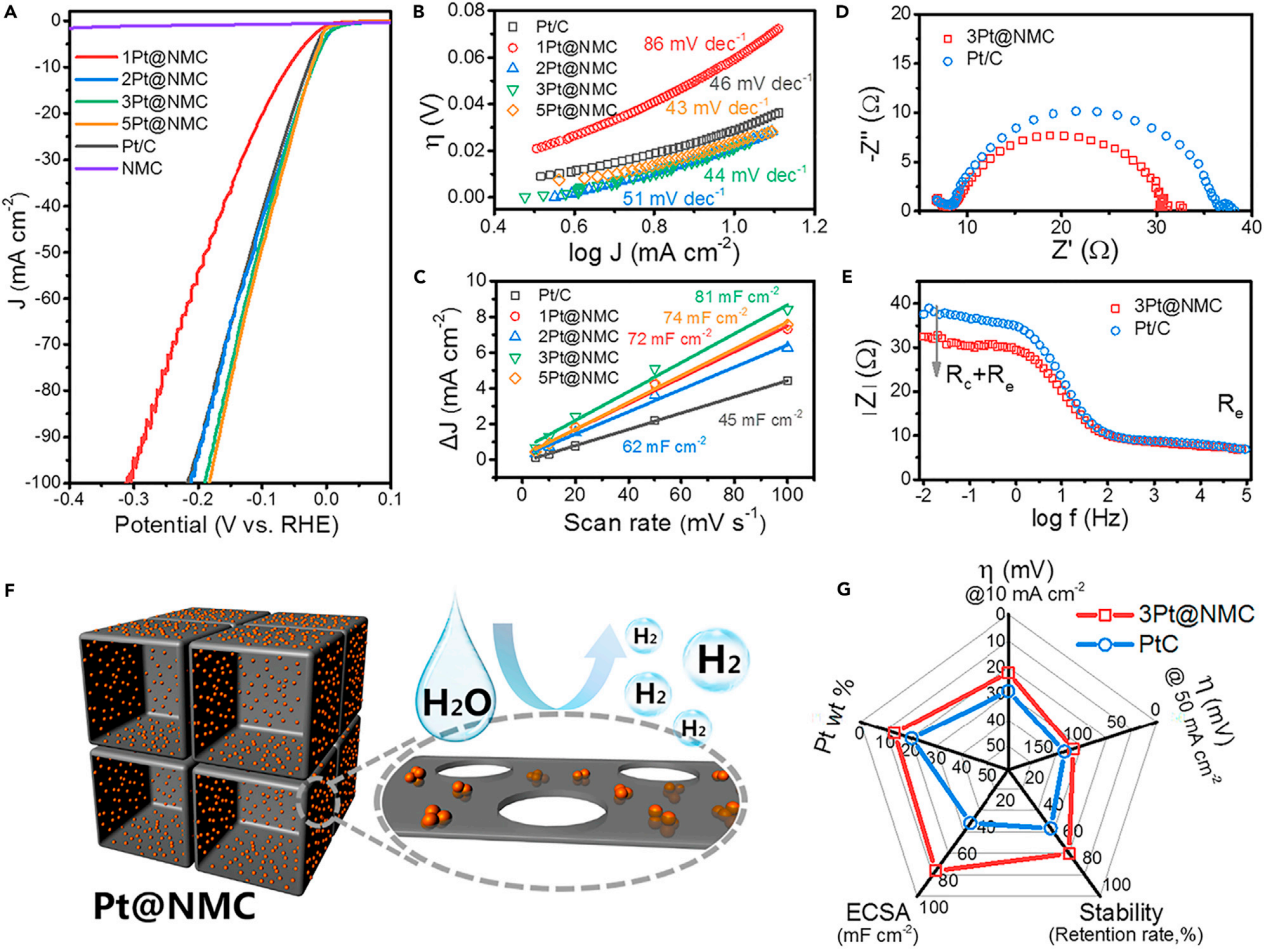
The catalytic properties of Pt@NMC in alkaline electrolyte (A) Linear sweep voltammetry (LSV) curves of Pt@NMC and the commercial Pt/C. (B) Tafel plots of Pt@NMC and the commercial Pt/C. (C) Capacitive currents vs. scan rates of Pt@NMC and the commercial Pt/C. (D) Nyquist plots of 3Pt@NMC and the commercial Pt/C. (E) Bode plots of |Z| vs frequency for 3Pt@NMC and the commercial Pt/C. (F) Schematic diagram of enhanced HER reaction of Pt@NMC. (G) Radar chart for comparing the comprehensive performances of 3Pt@NMC and the commercial Pt/C.

The electrocatalytic properties of Pt@NMC were evaluated in both alkaline and acidic solutions. For polarization curves tested in 1.0 M KOH, the catalytic activities of Pt@NMC samples gradually elevate with the increase of Pt content, yet such performance improvement shows the diminishing marginal benefit (Figure 3A). When the current density is 10 mA cm^−2^ (a common test standard), the overpotential of 3Pt@NMC is 22 mV lower than that of commercial Pt/C (29 mV), and the Pt load of the former is only two-thirds of the latter. At high current density (100 mA cm^−2^), the performances of Pt@NMC (*e.g.* 190 mV of 3Pt@NMC and 184 mV of 5Pt@NMC) still exceed their commercial comparison (218 mV of Pt/C). Tafel slope of 3Pt@NMC is calculated to be 44 mV dec^−1^, lower to that of Pt/C (46 mV dec^−1^), demonstrating the faster HER reaction kinetics of Pt@NMC (Figure 3B). Unsurprisingly, NMC exhibits the poor catalytic activity due to the absence of Pt. In the acidic electrolyte, Pt@NMC samples still have the high activity, such as achieving the overpotential of 125 mV at 100 mA cm^−2^ for 3Pt@NMC. Yet, in contrast with alkaline, the performances of Pt@NMC in 0.5 M H_2_SO_4_ do not exceed that of the commercial Pt/C (Figure S18). The Tafel slopes indicate that the catalytic kinetic process of Pt/C is faster than that of Pt@NMC in acidic system (Figure S19). On the whole, the enhancement effect of Pt@NMC catalyst is more prominent in alkaline solution which had the potential for industrial production (Holladay et al., 2009).

To explore the merits of Pt@NMC in alkaline electrolyte, the in-depth electrochemical analyses were adopted. Cyclic voltammetry (CV) curves at various scan rates within the non-Faraday potential zone were applied to calculate the electrochemical active area (ECSA) of Pt@NMC (Figure 3C) (Voiry et al., 2018). The double-layer capacitance (C_dl_) of 3Pt@NMC is 81 mF cm^−2^, which is the highest of various samples and almost double of the commercial Pt/C value (45 mF cm^−2^). The ultrafine Pt nanoclusters and the carbon support with large surface area contribute to such high C_dl_, corresponding to more active sites. In Nyquist plots recorded at the overpotential of 30 mV (vsRHE), both 3Pt@NMC and Pt/C present the typical semicircle for catalyzing HER (Figure 3D). As shown in the Bode plot of |Z| vs. log f (Figure 3E), 3Pt@NMC shows the similar impedance value with Pt/C in the high-frequency region, indicating that they have approximate impedance caused by the test system and the electrolyte (R_e_); in low-frequency region, the lower impedance of 3Pt@NMC indicates the faster charge transfer process (R_c_). Besides, the chronoamperometric (CA) tests identity that 3Pt@NMC retain 68% of the initial current density, yet the Pt/C only remain 49% after 10 h testing (Figure S20), proving the better stability of 3Pt@NMC. To sum, faster catalytic kinetics, larger electrochemical active area, and smaller charge transfer resistance contribute to the excellent catalytic performance of Pt@NMC in alkaline electrolyte (Figure 3F). Overall, we summarized the main indicators (*e.g.* activity, cost, stability) of 3Pt@NMC and the commercial Pt/C into a radar chart to demonstrate the comprehensive advantages of data-driven design catalysts (Figure 3G).

To analyze the relation of each factor and the overpotential, we performed a separate fitting analysis of the features and the overpotentials of Pt@NMC. As shown in Figure S21, the Pt content (Pt_wt) shows a certain correlation with the overpotential since their correlation coefficient reached 0.54, which is higher than that of N_doped and surface_area. Meanwhile, by studying the control group samples, we illustrate the complexity of the Pt/C system (Figures S22 and S23). Specifically, Control-1 has a higher Pt content (11.6 wt %) than that of 2Pt@NMC (7.7 wt %). Yet, the surface area (488.9 m^2^ g^−1^) and N-doped content (8.2 wt %) of Control-1 are lower than that of the corresponding features of 2Pt@NMC (601.2 m^2^ g^−1^ and 10.0 wt %). Tested in KOH, 2Pt@NMC and Control-1 show similar catalytic activities. As a comparison, 2Pt@NMC and Control-2 have similar Pt content. However, the surface area and N-doped content of Control-2 are lower than that of 2Pt@NMC, leading to its weaker catalytic performance. These data prove the complex characteristic of the catalysts with various features. Even so, the correlation coefficient between Pt content and overpotential still exceeds the values of other factors, which is in line with the results of the ML analysis.

### Prediction and data-feedback

**Figure 4:**
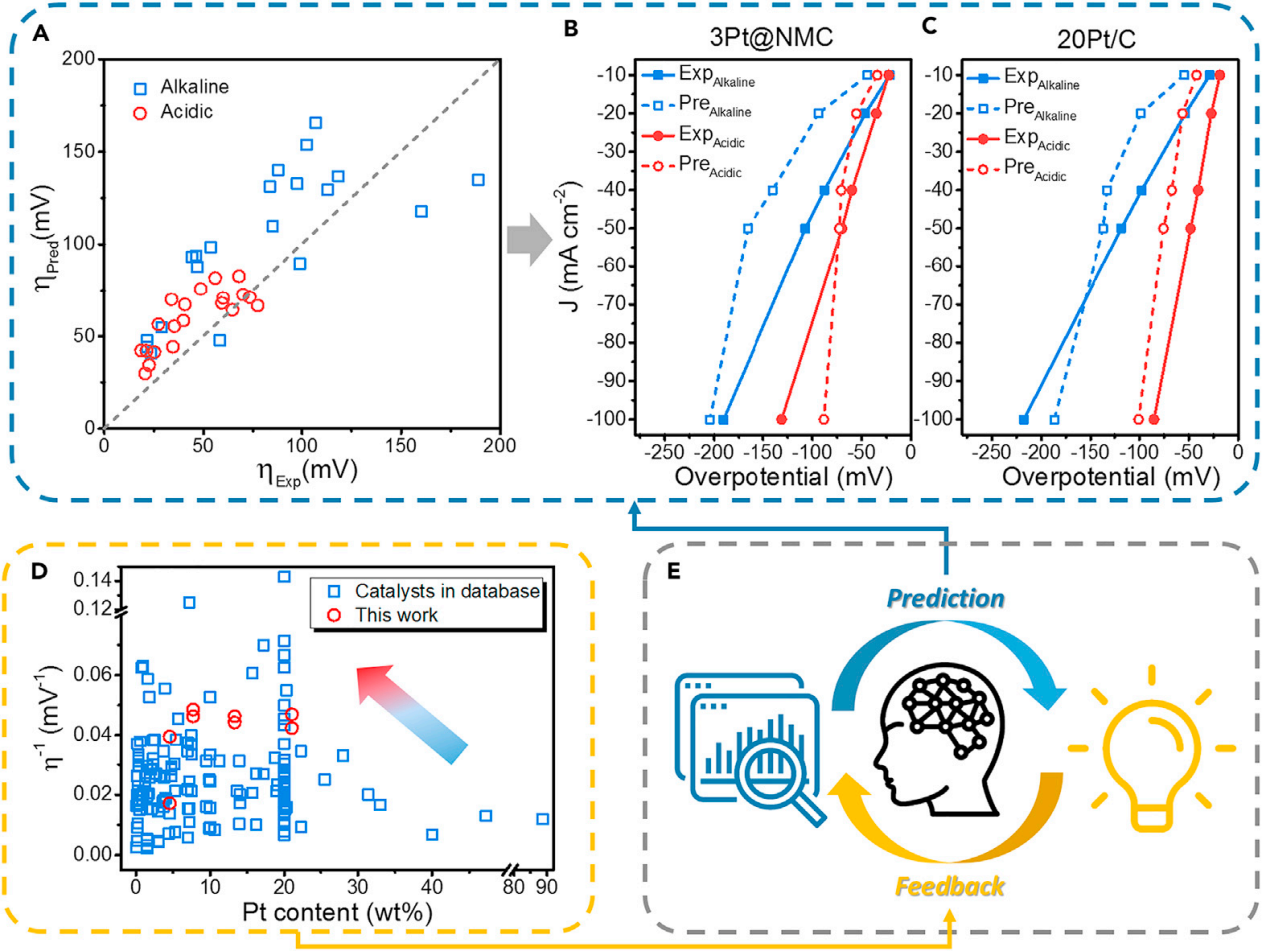
The closed-loop of the data-driven catalyst design (A) Comparison of the catalytic overpotential predicted by ML and the actual value from experiment tests for Pt@NMC and the commercial Pt/C. (B) Comparison of predicted LSV curves and actual data for 3Pt@NMC. (C) Comparison of predicted LSV curves and actual data for the commercial Pt/C. (D) Performance comparison for the data from the original database and Pt@NMC. (E) Schematic diagram of research closed-loop from data mining to catalyst evaluation.

Now that we have the data of the prepared materials, the ML methods could be conducted again. By entering the characteristics of Pt@NMC, the algorithm optimized by TPOT could predict their corresponding catalytic performance (Figure 4A). In general, the predicted η_Pred_ and the actual η_Exp_ were roughly linear, but there is a certain degree of errors (Table S7). In this study, the experimental and predicted values of Pt@NMC are close in the acid electrolyte. For example, for 3Pt@NMC in acid electrolyte (pH = 0.3), the error is 3.9% at 10 mA cm^−2^ and 8.9% at 50 mA cm^−2^. In contrast, in alkaline electrolytes, the error is relatively large (Figure 4B). There are two reasons for this difference. On the one hand, there are less alkaline data in the original database, and the lack of data makes the algorithms have a large error in the prediction results. On the other hand, the Pt@NMC samples do show above-average performance in alkaline solution, and they can use less Pt loading and achieve better catalytic performance than the commercial Pt/C. If we observe the commercial Pt/C, the gap between the real and the prediction is not large, for example, the predicted value (134 mV) is not far from the measured value (118 mV) at 50 mA cm^−2^ (Figure 4C), meaning that the commercial catalyst is close to the average properties of the Pt-C catalyst system. In addition to these two reasons, the limitations of the ML model also introduce errors. Because only six features were selected, our model cannot cover all the material characteristics to analyze the Pt/C catalysts.

The whole picture of Pt/C HER catalysts was plotted in Figure 4D, which contained the data of over 200 Pt-based catalysts and Pt@NMC. The vertical and horizontal axes in the plot are the reciprocal of the overpotential and the Pt content, roughly reflecting the catalytic activity and the cost of the materials. Specifically, the data-driven designed catalysts (Pt@NMC) are in the upper left corner of all data, *i.e.* it had the merits of both higher catalytic activity and lower cost. Furthermore, we combined the data of this work with the original database and analyzed the importance of each feature. The recalculated SHAP values have some changes (Figure S24), such as that the importance of surface_area has increased slightly. This phenomenon suggests that the new data are constantly improving the model, thus enhancing the algorithm's understanding on the catalytic process. All these are brought together and connected by ML to form a closed-loop research (Figures 1A and 4E). This closed-loop framework can continuously improve its operating efficiency by feedback and updates, thereby accelerating the development of high-performance catalysts.

### Conclusion

In this work, we developed a data-driven strategy to design high-performance electrocatalysts for H_2_ evolution. Specifically, a Pt/C HER catalyst database was built by collecting data from the previous reports, and the ML model optimized by TPOT was applied to investigate the catalyst system. By using SHAP to open the “black box” of the algorithm, the impacts of factors were quantitatively analyzed to form guidance for catalyst synthesis. Among various features, we found that the Pt content and Pt size have the greatest influence on the performance of catalysts. Based on the ML outcome, a nitrogen-doped mesoporous carbon network prepared by salt template was used as the confined support to fabricate the content-controllable and size-controllable Pt nanoclusters. The features of the obtained Pt@NMC were in line with the intelligent insights and showed superior catalytic properties in alkaline electrolyte. As a result, we built a closed-loop framework of catalyst development, consisting of data mining, ML analysis, controllable synthesis, and performance evaluation. Furthermore, the framework is available for anyone to add their research data and improve their own catalysts by using our open-source database. By continuously introducing more dimensions and more quantities of data, the researchers can reveal a more comprehensive and accurate importance ranking among the features in the catalyst systems. Overall, this work provided an example of a data-driven research paradigm to design optimized catalysts, which is hopefully extended to the R&D of other state-of-art materials.

### Limitations of the study

In this work, the analysis given by ML is not detailed, because the current amount of data and the features are not enough to support a precise model. Thus, we applied the TPOT and SHAP algorithms to rank the importance of several features. Although the experimental tests prove that the importance of ranking is helpful, it is better to introduce more data and more complete features to give full play to the potential of ML. Furthermore, the prepared samples perform better in alkaline electrolytes than in the acid, which requires follow-up experiments and in-depth mechanism research.

## STAR★Methods

### Key resources table

**Table:** 

REAGENT or RESOURCE	SOURCE	IDENTIFIER
**Chemicals, peptides, and recombinant proteins**		

Commercial Pt/C	Hesen	CAS#7440-06-4
Sodium chloride	Kermel Reagent	CAS#7647-14-5
Anhydrous glucose	Kermel Reagent	CAS#50-99-7
Sodium silicate	Kermel Reagent	CAS#1344-09-8
Chloroplatinic acid	Kermel Reagent	CAS#16941-12-1(81507)
Urea	Guangfu	CAS#57-13-6
Potassium hydroxide	Meryer	CAS#1310-58-3(82002)
N-N Dimethylformamide	Aladdin Reagent	CAS#68-12-2(33627)
Sulfuric acid	Yuanli	CAS#7664-93-9(81007)
Nafion	Sigma-Aldrich	CAS#31175-20-9

**Software and algorithms**		

Python version 3.10.0	Python Software Foundation	https://www.python.org/
WebPlotDigitizer	Ankit Rohatgi	https://apps.automeris.io/wpd/
Code for ML models	This paper	https://github.com/Shan-Zhu/ML-HER-PtC

**Deposited data**		

Data S1. Database of Pt/C catalysts, Related to STAR Methods.	This paper	https://github.com/Shan-Zhu/ML-HER-PtC/blob/main/SI-All_PtC_Database.csv
Data S2. Dataset of Pt/C catalysts used in ML models, Related to Figure 1.	This paper	https://github.com/Shan-Zhu/ML-HER-PtC/blob/main/SI-ML_PtC_Database.csv

### Resource availability

#### Lead contact

Further information and request for resources should be directed to the lead contact, Shan Zhu (shanzhu@tju.edu.cn)

#### Materials availability

This study did not generate new unique reagents.

### Method details

#### Data collection and machine learning

To build the database, we collected hundreds of published papers about Pt/C catalysts for HER. Among them, over 200 sets of data were manually extracted, including the features of Pt, the physical and chemical features of carbon support and the test system. All of these data were available in the file of Data S1. Database of Pt/C catalysts, Related to STAR Methods. Then, we chose the reported samples with comprehensive characteristics of the selected features, and further extracted their actual test performance under various current densities with the help of WebPlotDigitizer, thus forming a new database of Data S2. Dataset of Pt/C catalysts used in ML models, Related to Figure 1. Using such database, we applied the algorithms of TPOT (tree-based pipeline optimization tool) to analyze the Pt/C-HER catalyst system. The optimized model figured out by TPOT was the Gradient Boosting Regressor. After determining the optimization model, to understand the contribution of each feature in the model, the TreeExplainer in the SHAP library was called to calculate the Shapley value of each feature. The brief overviews for these algorithms were in the supplemental information. All ML methods were conducted in Python, and the corresponding codes were available at https://github.com/Shan-Zhu/ML-HER-PtC.

#### Materials synthesis

Preparation of N-doped mesoporous carbon nanosheet network (NMC): Firstly, anhydrous glucose (1.25 g), NaCl (20 g), Na_2_SiO_3_ (1.25 g), urea (1.25g) and deionized water (100 ml) were stirred together for 2 h. Next, the mixed solution was frozen in -40°C for 24 h and freeze-dried at -65°C in vacuum for 24 h to obtain composite powders. Then, the composite powders were annealed at 650°C (heating rate of 8°C min^-1^) for 2 hours in Ar (250 mL min^-1^) in a tube furnace. The heated powders were washed out the NaCl and Na_2_SiO_3_ templates with deionized water, and then dried at 60°C under vacuum overnight to obtain NMC.

Preparation of xPt@NMC (x=1, 2, 3, 5): 10 mg of NMC were dispersed in 5 mL of 0.01x M H_2_PtCl_6_ solution by sonication for 30 min, and then stood for 24 h. The obtained mixture was separated by suction fitration and dried overnight at 60°C. Then, the dried mixture was rapidly heated to 400°C (heating rate of 100°C min^-1^), hold for 10 min in Ar (200 mL min^-1^) and cooled to room temperature. The obtained product were noted as xPt@NMC.

Preparation of Control-1 and Control-2: When preparing the carbon matrix of Control-1, the input amount of urea was changed to 0.625 g. For Control-2, the added urea was 0.125 g. Other processes were the same as 3Pt@NMC.

#### Characterizations

The morphology of as-prepared samples were studied by using transmission electron microscopy (TEM), high-resolution TEM (HRTEM), scanning TEM (STEM) transmission electron microscopy on aFEITecnaiG2F20 TEM and Scanning electron microscopy (SEM) on a Hitachi S4800. The crystalline structures of samples were examined by X-ray diffraction (XRD) on a Rigaku D/max diffractometer with Cu Ka radiation. X-ray photoelectron spectroscopy (XPS) measurements were carried out on a PHI 5000 VersaProbe using an Al Kα X-ray source. Brunauer-Emmett-Teller (BET) specific surface and porosities of the samples were acquired by a Micromeritics ASAP 2020 analyzer using nitrogen adsorption and desorption. Raman spectrum were performed on a LabRAMHR Raman spectrometer by applying an Ar ion laser source at the laser excitation of 514.5 nm. Thermogravimetric analysis (TGA) was recorded on NETZSCHSTA449F3 at a heating rate of 10°C min^-1^ in air from room temperature to 800°C.

#### Electrochemical test

All electrocatalytic activity was tested on an Ivium-n-Stat workstation using a typical three-electrode system at the room temperature. The graphite rod electrode acted as the counter electrode. The saturated calomel electrode (SCE) and Ag/AgCl electrode (SSCE) served as the reference electrode in 1.0 M KOH and 0.5 M H_2_SO_4_, respectively. The glassy carbon electrode (3 mm diameter) loading 5 μL ink was used as the working electrode. The inks were prepared by dispersing 2.5 mg of catalyst samples in 480 μL of DMF and 20 μL of 5% Nafion for 20 min by sonication. The HER tests were carried out using LSV with a scan rate of 5 mV s^-1^. Double-layer capacitances (C_dl_) were conducted by CV scanning within the potential window of 0.068∼0.268 V vsRHE in 1.0 M KOH, and 0.097∼0.197 V vsRHE in 0.5 M H_2_SO_4_. The ideal specific capacitance was generally considered to be 60 μF cm^-2^. The Tafel slopes were calculated by the LSV curves. Electrochemical impedance spectroscopy (EIS) date were measured with the AC voltage amplitude of 7 mV. The stability of the catalysts were evaluated by an amperometrici-t curve tests at the current density of 10 mA cm^-2^ for 10 h.

## Data Availability

• The data of Pt/C catalysts databases have been deposited at https://github.com/Shan-Zhu/ML-HER-PtC and are publicly available as of the date of publication. Other data reported in this paper will be shared by the lead contact upon request. • All original code has been deposited at https://github.com/Shan-Zhu/ML-HER-PtC and is publicly available as of the date of publication. • Any additional information required to reanalyze the data reported in this paper is available from the lead contact upon request.

## Supplemental information

Document S1. Figures S1–S24 and Tables S1–S7

Data S1. Database of Pt/C catalysts, related to STAR Methods

Data S2.Dataset of Pt/C catalysts used in ML models, related to Figure 1

## Footnotes

Supplemental information can be found online at https://doi.org/10.1016/j.isci.2021.103430.
