# Supplementary material for: Data-driven design and controllable synthesis of Pt/carbon electrocatalysts for H2 evolution
Source: iScience. 2021 Nov 13;24(12):103430. doi: 10.1016/j.isci.2021.103430 (PMC8637634; doi:10.1016/j.isci.2021.103430)
Supplement: Document S1. Figures S1–S24 and Tables S1–S7 [file mmc1.pdf]

iScience, Volume *24*

Supplemental information

Data-driven design and controllable synthesis of Pt/carbon electrocatalysts for H_2 evolution

Anhui Zheng, Yuxuan Wang, Fangfei Zhang, Chunnian He, Shan Zhu, and Naiqin Zhao

SUPPLEMENTAL INFORMATION

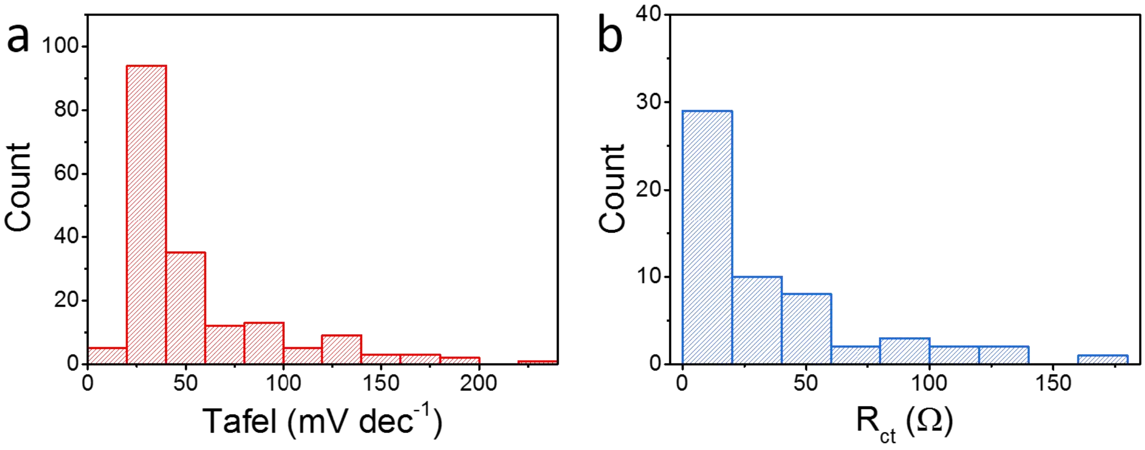

Figure S1. The distribution of some of the data in the database. Related to Figure 1.

(A) The statistical distribution of Tafel slope.

(B) The statistical distribution of R_{ct}.

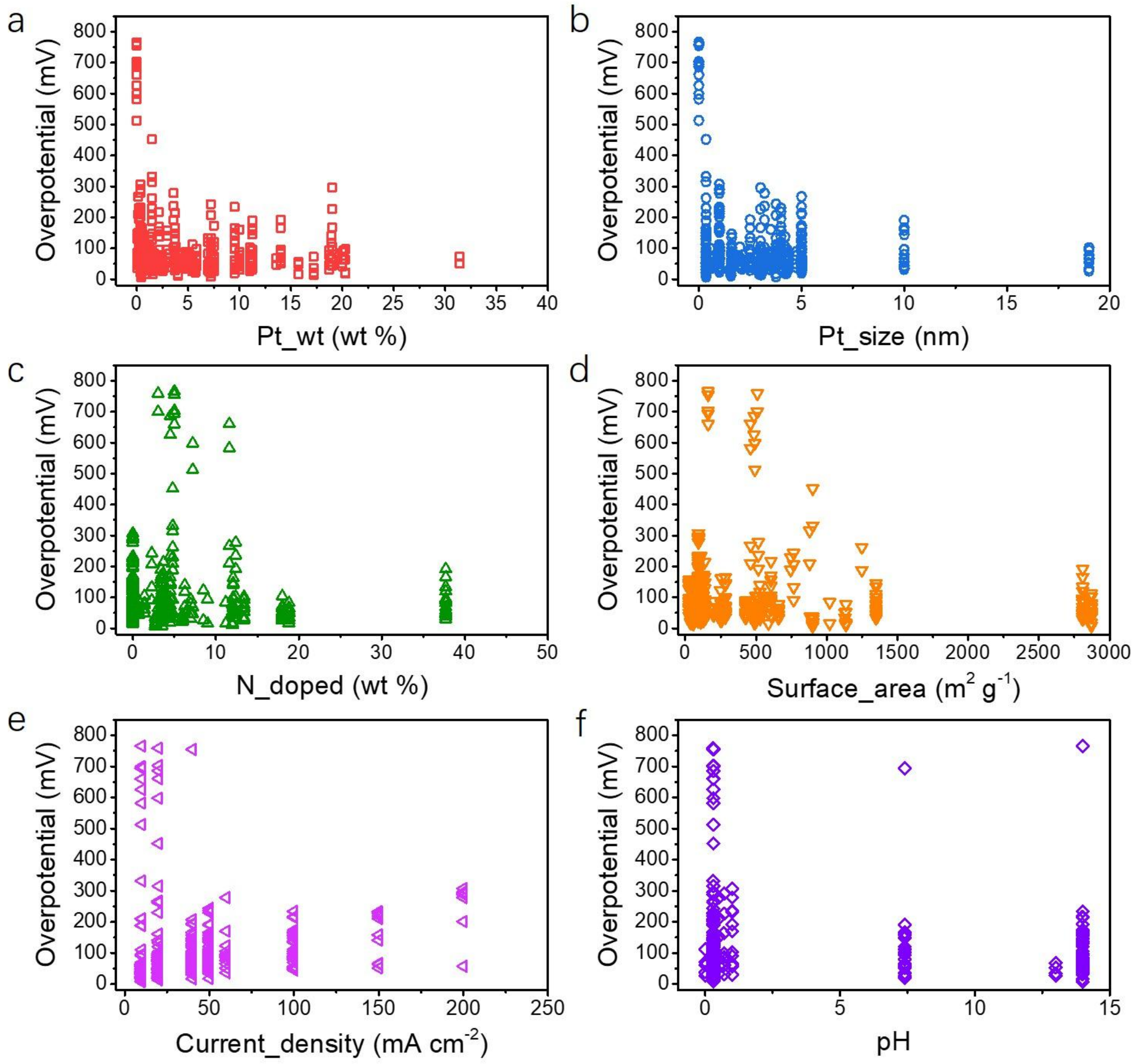

Figure S2. The plot of the features and the overpotential. Related to Figure 1.

(A) The plot of Pt_wt and the overpotential.

(B) The plot of Pt_size and the overpotential.

(C) The plot of N_doped and the overpotential.

(D) The plot of Surface_area and the overpotential.

(E) The plot of Current_density and the overpotential.

(F) The plot of pH and the overpotential.

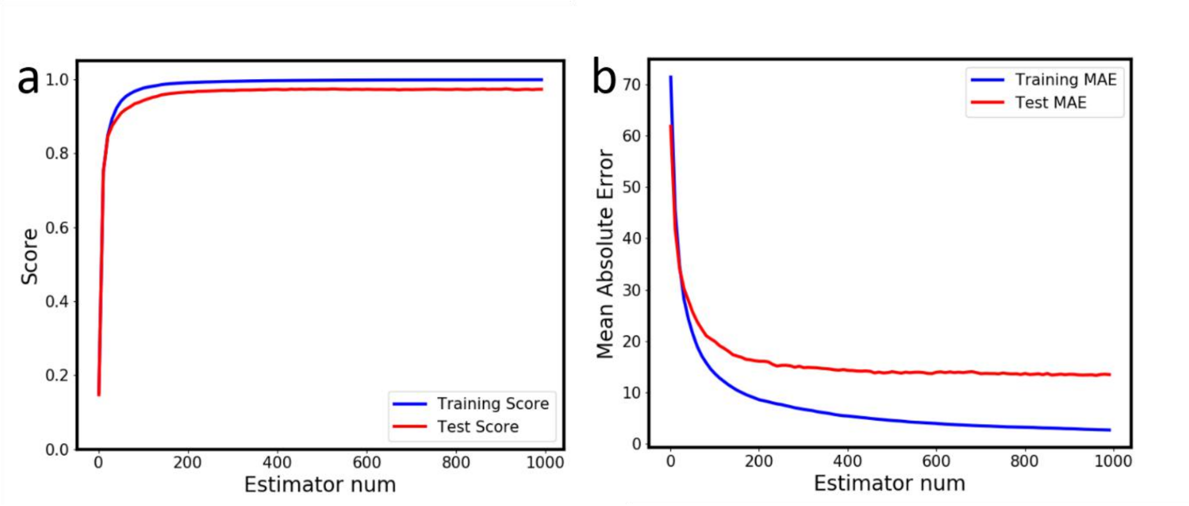

Figure S3. Algorithm running performance. Related to Figure 1.

(A) The score in training/testing dataset during the training progress using the TPOT selected Gradient Boosting Regressor model.

(B) MAE value in training/testing dataset during the training progress using the TPOT selected Gradient Boosting Regressor model.

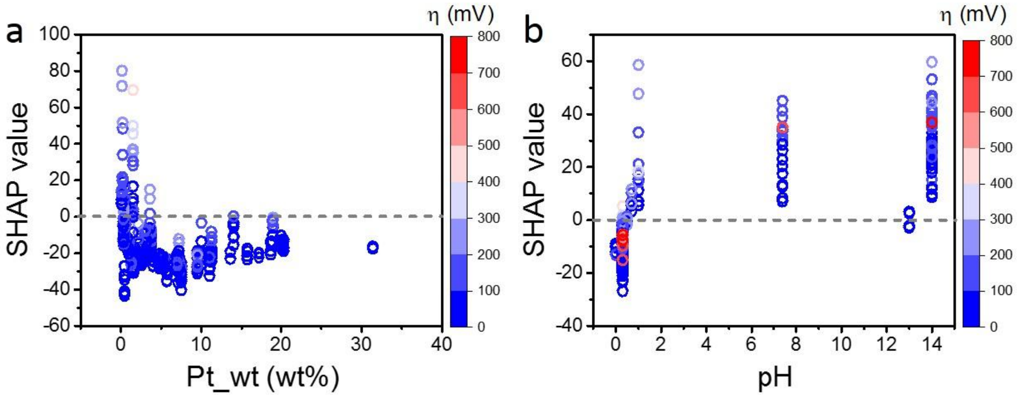

Figure S4. The SHAP value of the features. Related to Figure 1.

(A) The SHAP value of Pt_wt.

(B) The SHAP value of pH.

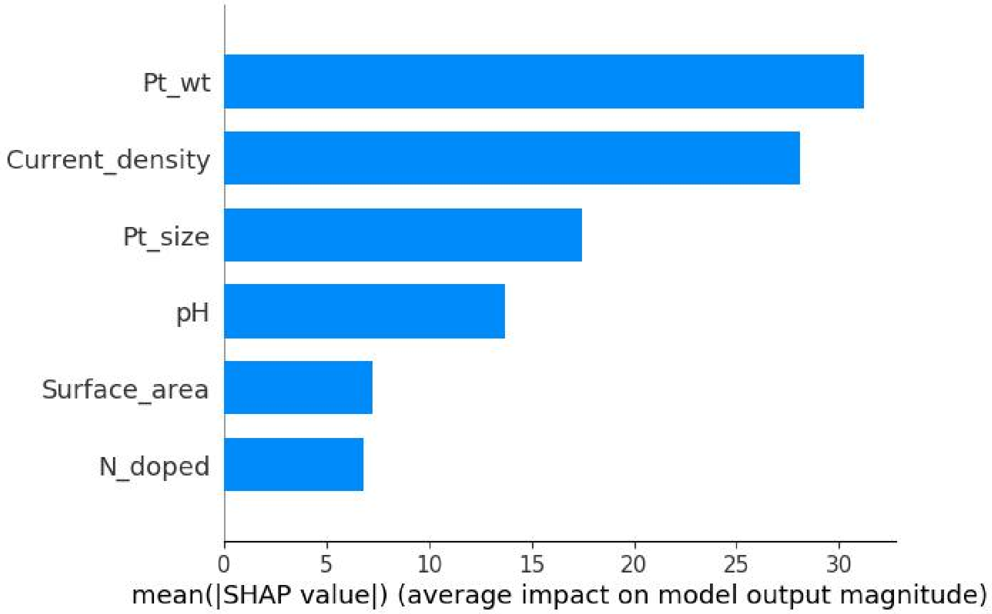

Figure S5. The average of |SHAP value| for each feature. Related to Figure 1.

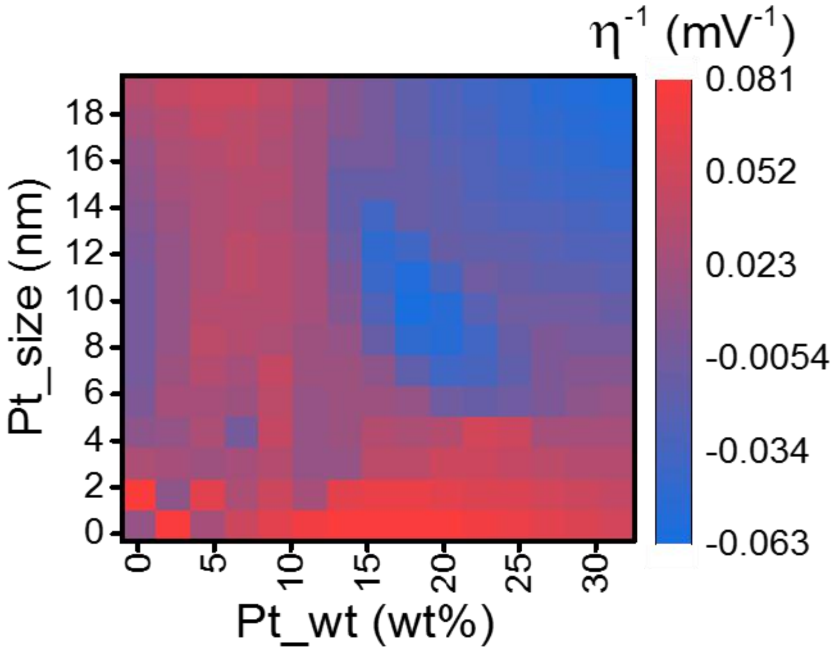

Figure S6. The Heatmap with the program automatically generated full range. Related to Figure 1.

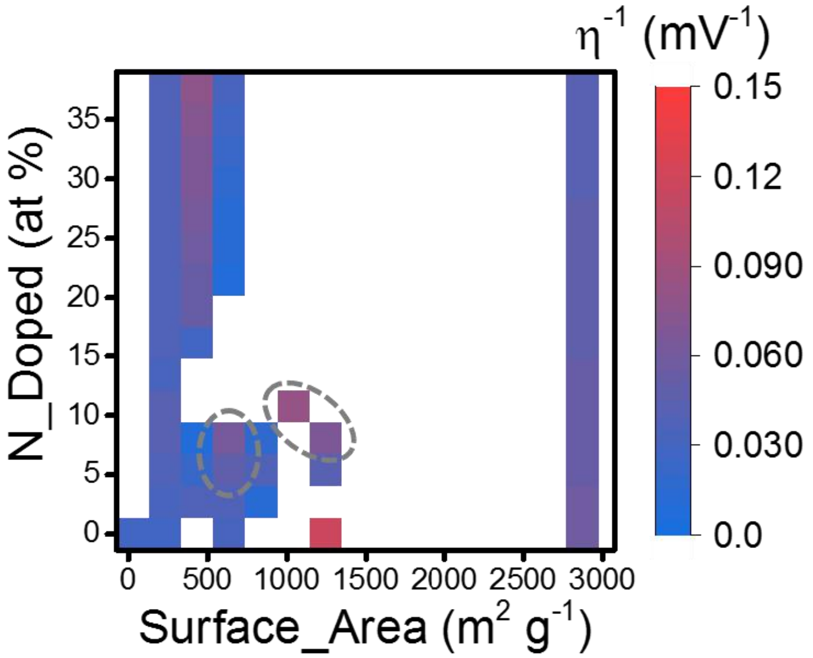

Figure S7. The Heatmap of N_doped and Surface_Area on η^{-1}. Related to Figure 1.

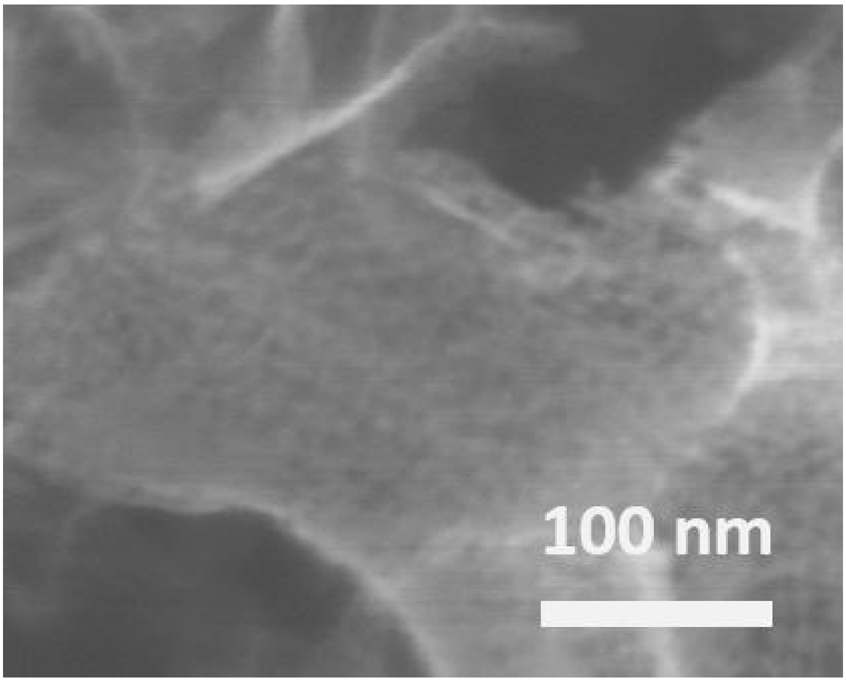

Figure S8. SEM image of the mesopores in NMC. Related to Figure 2.

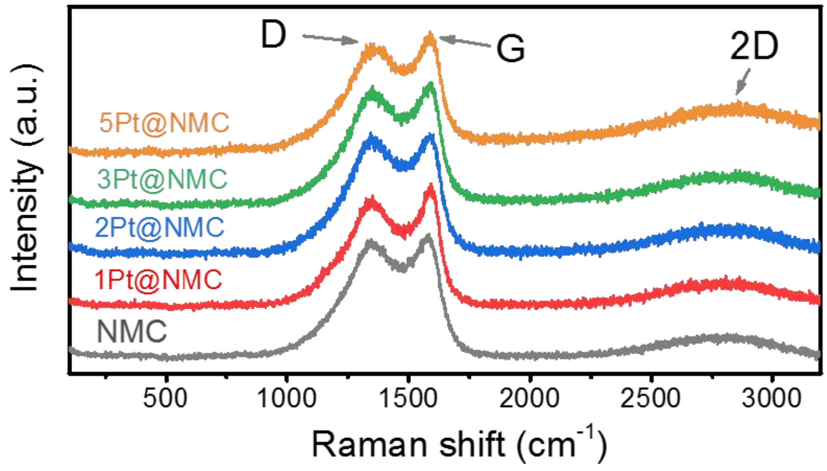

Figure S9. Raman results of Pt@NMC and NMC. Related to Figure 2.

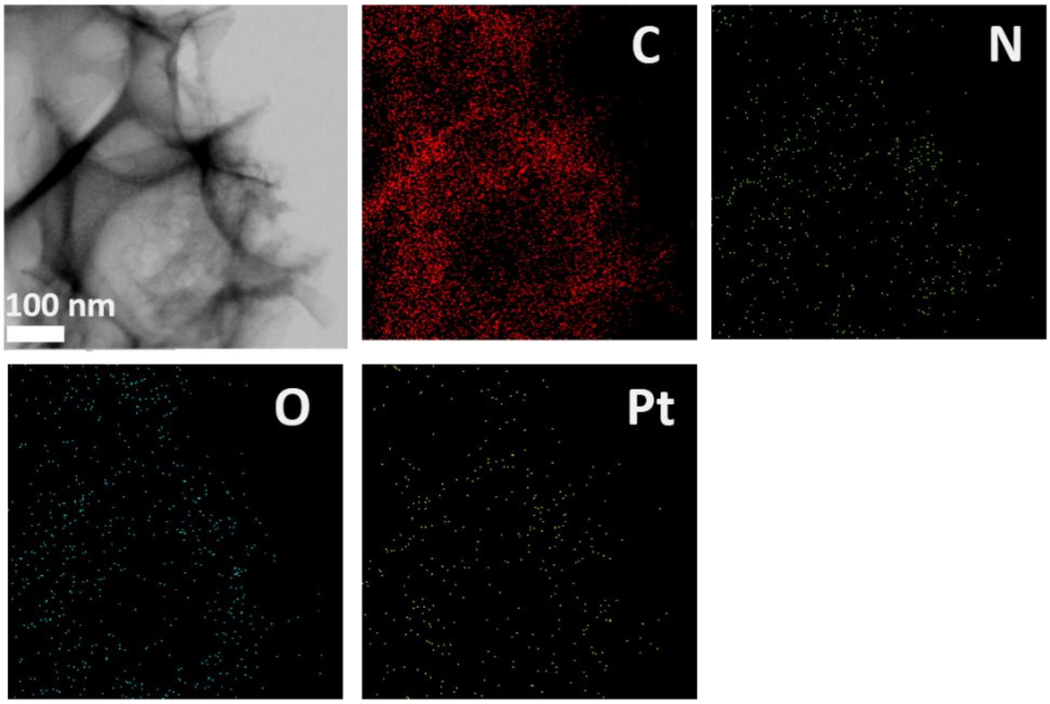

Figure S10. STEM of 3Pt@NMC showing its elements distribution. Related to Figure 2.

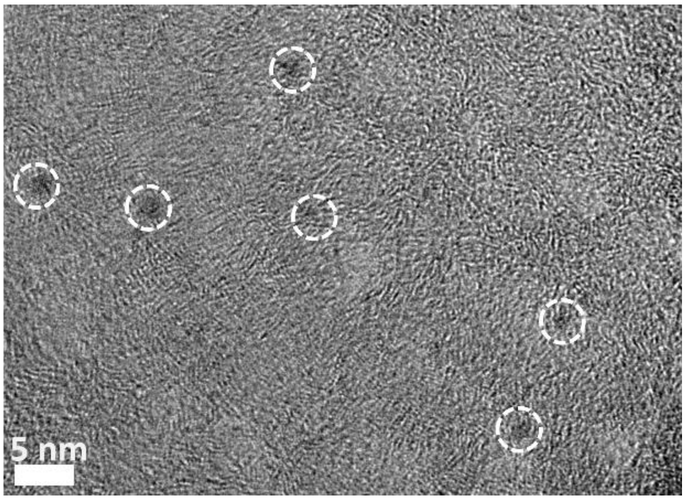

Figure S11. HETEM of 3Pt@NMC. Related to Figure 2.

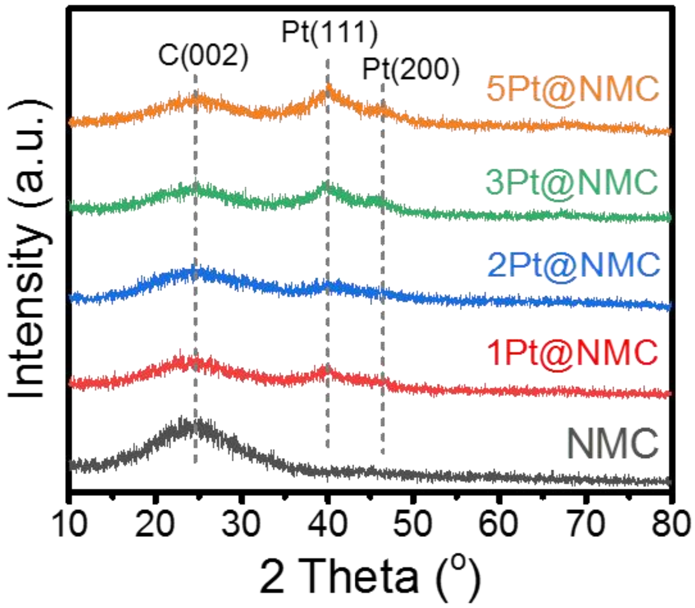

Figure S12. XRD results of Pt@NMC and NMC. Related to Figure 2.

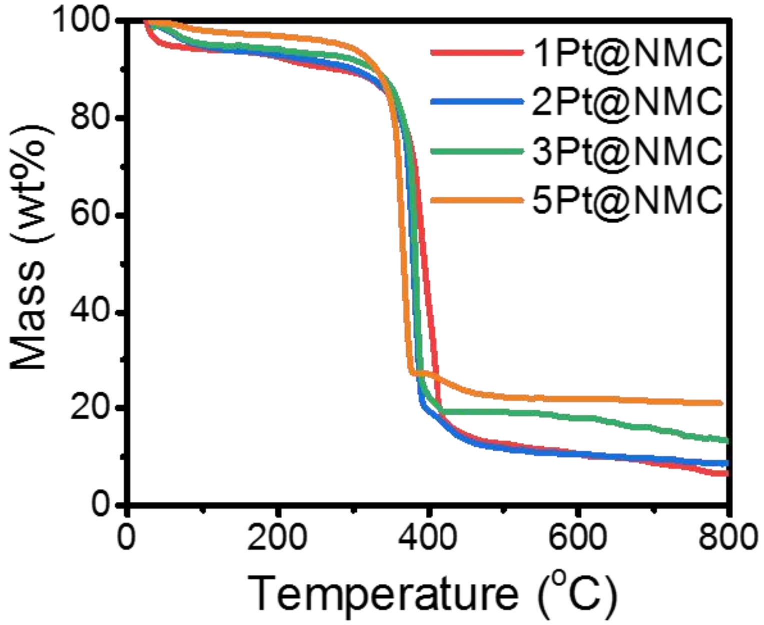

Figure S13. TGA results of Pt@NMC samples. Related to Figure 2.

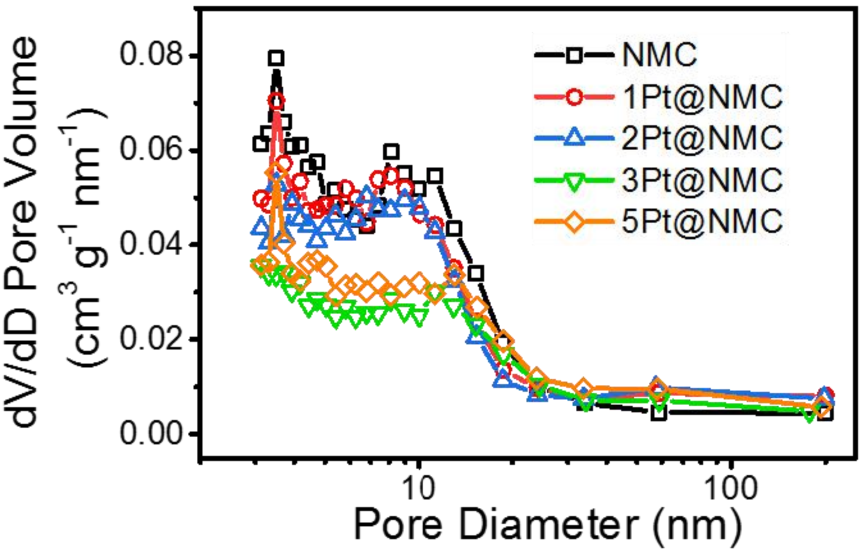

Figure S14. Pore size distribution of Pt@NMC and NMC, which were analyzed by BJH method. Related to Figure 2.

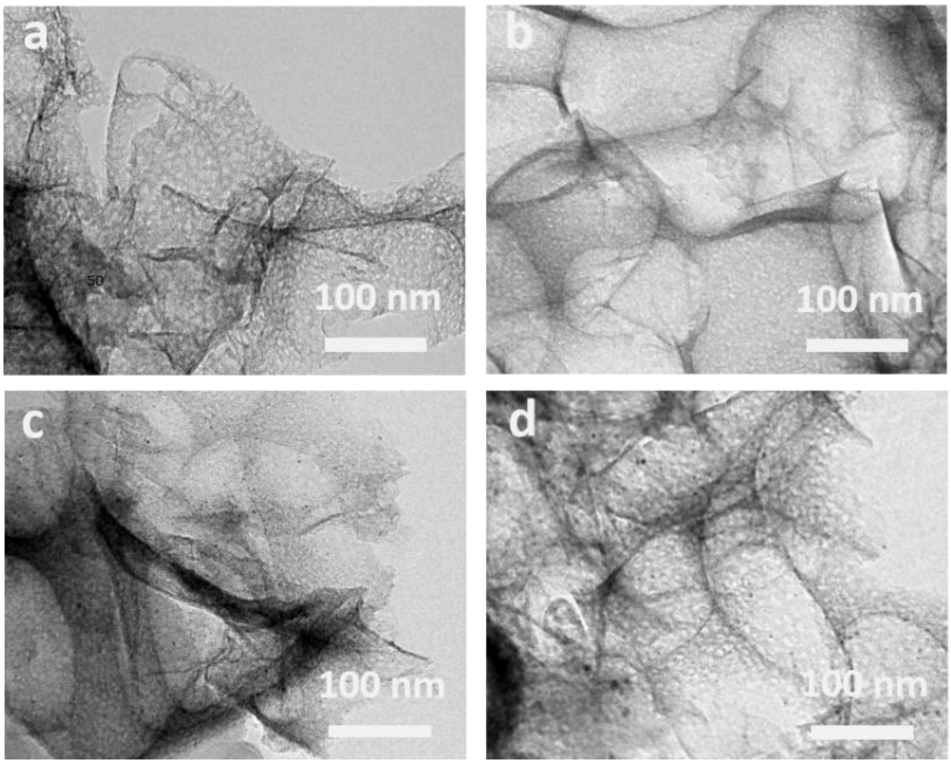

Figure S15. TEM image of Pt@NMC. Related to Figure 2.

(A) 1Pt@NMC.

(B) 2Pt@NMC.

(C) 3Pt@NMC.

(D) 5Pt@NMC.

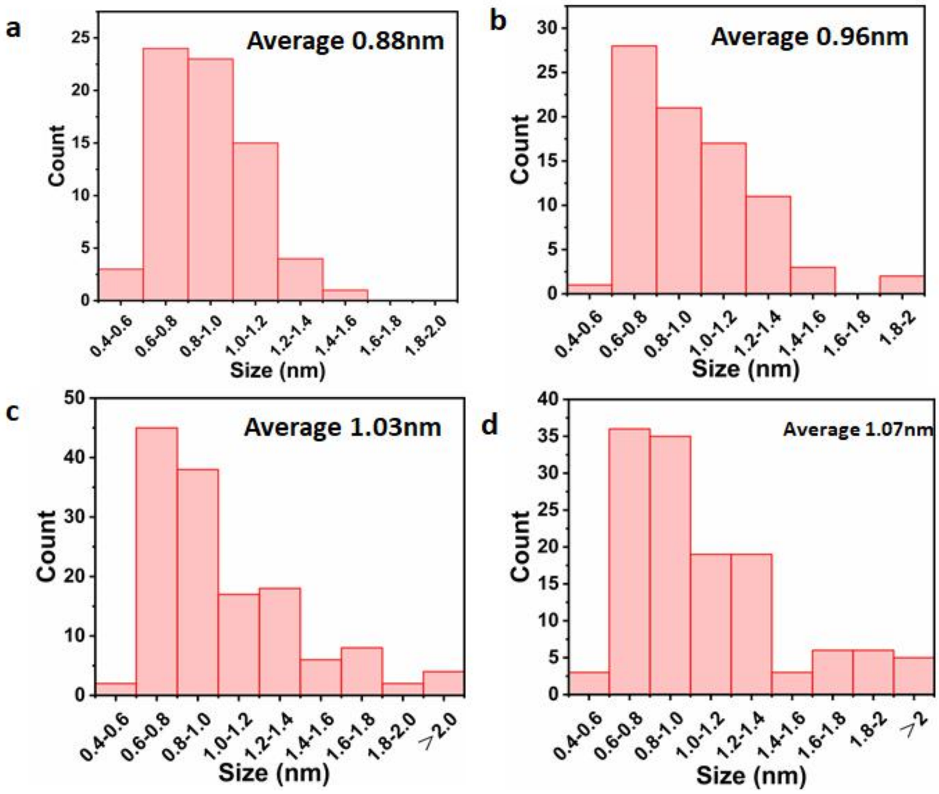

Figure S16. Pt particle size statistics of Pt@NMC samples. Related to Figure 2.

(A) 1Pt@NMC.

(B) 2Pt@NMC.

(C) 3Pt@NMC.

(D) 5Pt@NMC.

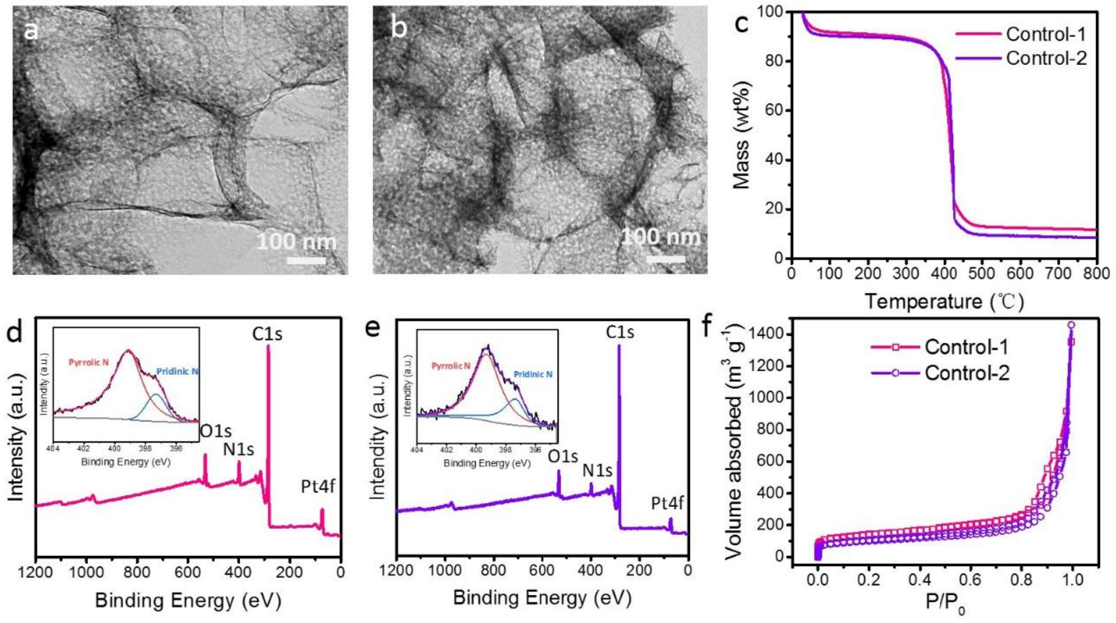

Figure S17. Characterization of control group samples. Related to Figure 2.

(A) TEM image of Control-1.

(B) TEM image of Control-2.

(C) TG results of Control-1 and Control-2.

(D) XPS result of Control-1.

(E) XPS result of Control-2.

(F) N_2 adsorption-desorption isotherms of Control-1 and Control-2.

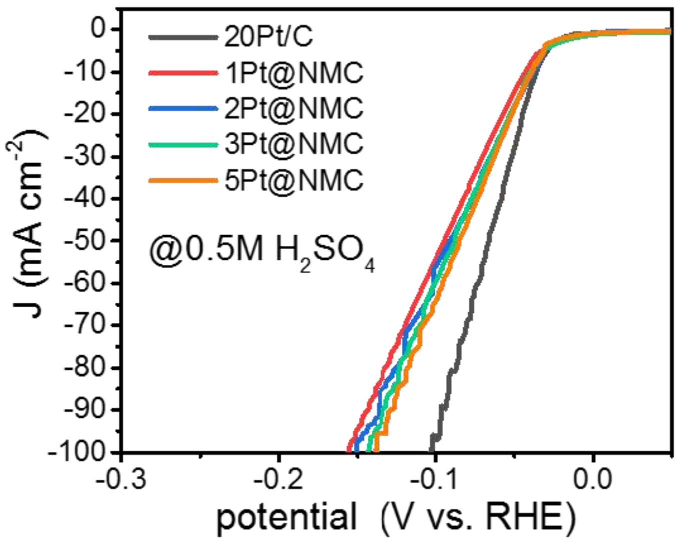

Figure S18. LSV curves of Pt@NMC and commercial Pt/C in 0.5M H_2SO_4. Related to Figure 3.

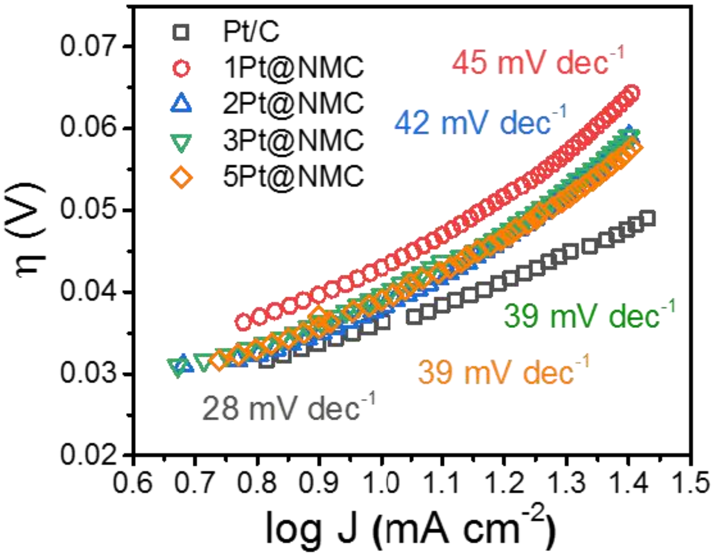

Figure S19. Tafel slopes of Pt@NMC and commercial Pt/C in 0.5 M H_2SO_4. Related to Figure 3.

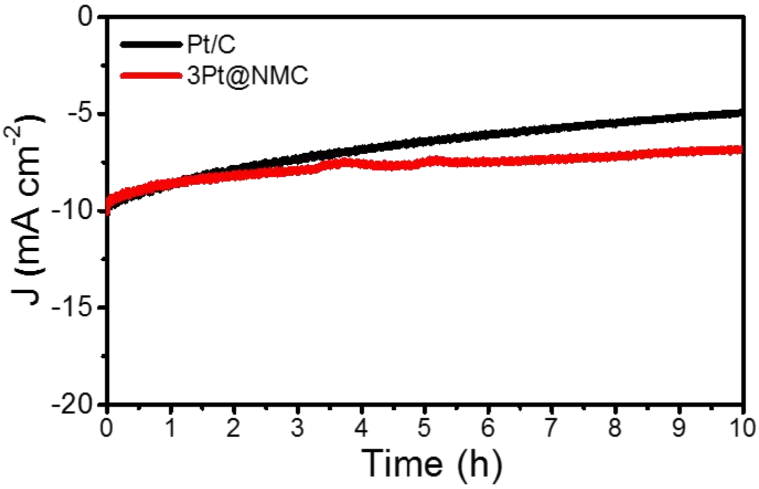

Figure S20. The stability test of 3Pt@NMC and commercial Pt/C. Related to Figure 3.

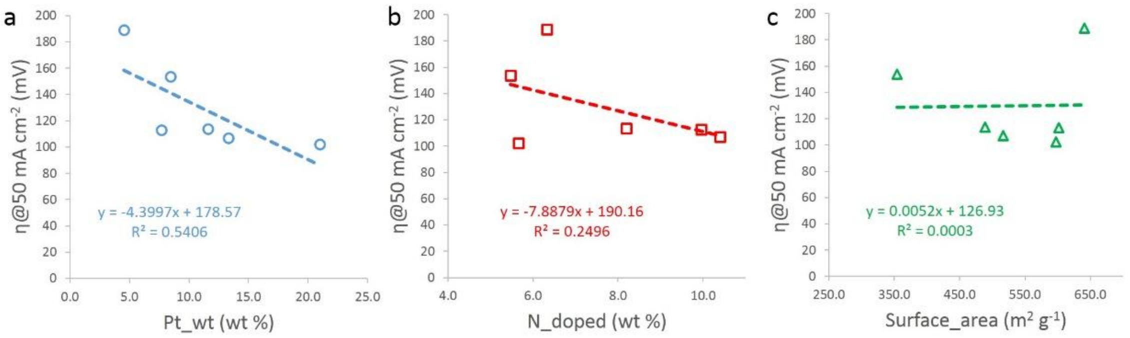

Figure S21. The single feature vs. the overpotential measured at 50 mA cm^{-2} in the alkaline solution. The data is fitted, and the formula are written in the corresponding figures. Related to Figure 3.

(A) Pt_wt.

(B) N_doped.

(C) Surface_area.

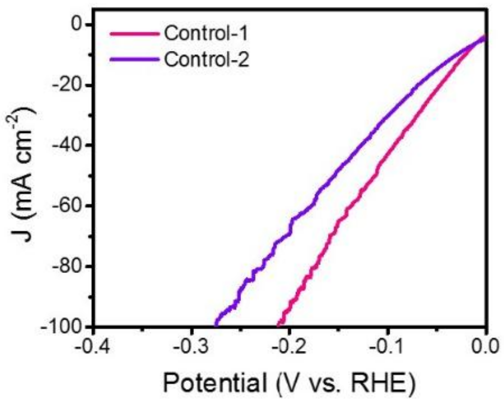

Figure S22. LSV curves of the Control-1 and Control-2 in 1M KOH. Related to Figure 3.

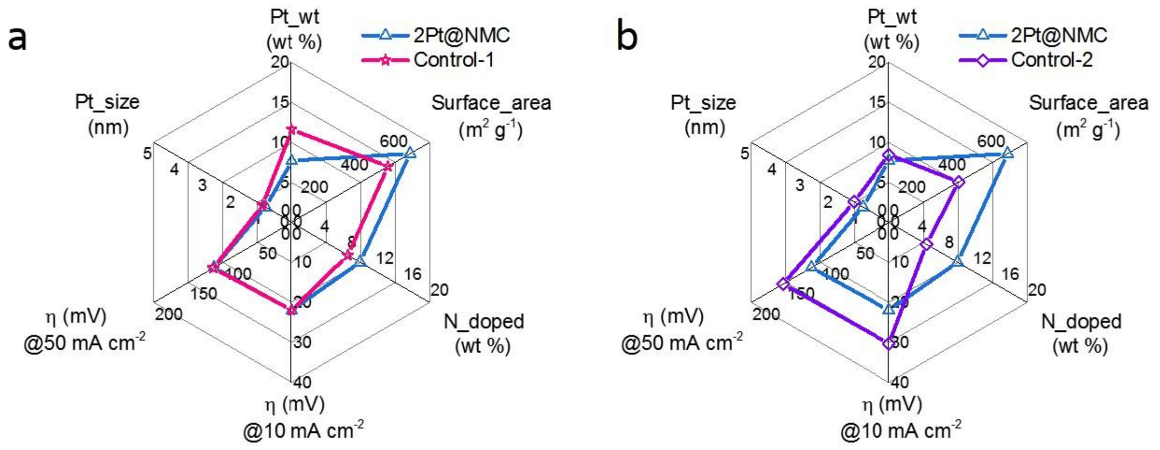

Figure S23. Comparison of 2Pt@NMC and the control samples (A) Control-1 and (B) Control-2. The comparison factors include Pt content, Pt size, specific surface area, N-doped content and the overpotential under different current densities. Related to Figure 3.

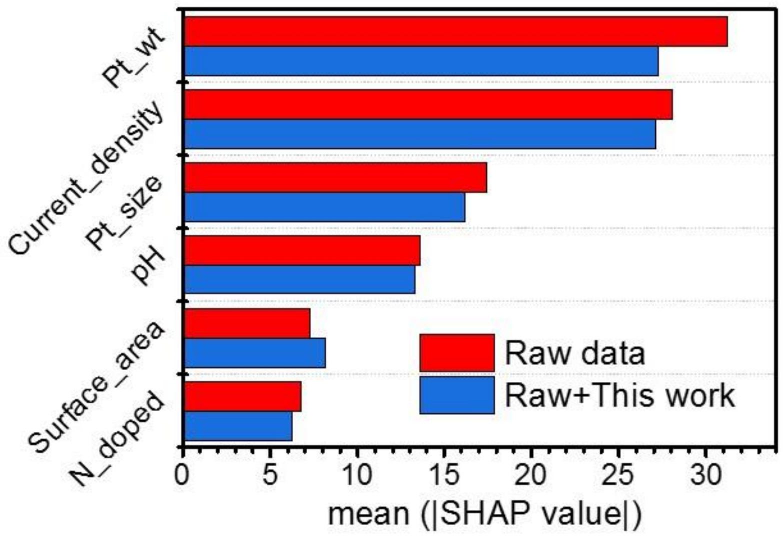

Figure S24. The recalculated SHAP after adding the data of this work in the original database. Related to Figure 4.

Table S1. Optimized main parameters of TPOT. Related to Figure 1.

Parameters	Value
Train/Test split ratio	0.8/0.2
Selected ML method by TPOT	Gradient Boosting Regressor
Random_state	99
Alpha	0.75
Learning_rate	0.1
Loss function	Least Squares Regression
Max_depth	8
Max_features	0.6
Min_samples_leaf	1
Min_samples_split	2
Subsample	0.7

Table S2. The evolution for the ML methods on the training/test dataset. Related to Figure 1.

	Value
Training r^2 Score	0.99
Test r^2 Score	0.97
Training Mean Squared Error	1.898
Test Mean Squared Error	338
Training Mean Absolute Error	0.8498
Test Mean Absolute Error	13.74

Table S3. The results of K-fold cross-validation. Related to Figure 1.

	K-fold (K=3)				
r^2 score	0.975	0.963	0.943		
	K-fold (K=4)				
r^2 score	0.979	0.940	0.947	0.974	
	K-fold (K=5)				
r^2 score	0.969	0.958	0.957	0.977	0.973
	K-fold (K=10)				
r^2 score	0.972	0.957	0.959	0.975	0.973
	0.977	0.931	0.973	0.884	0.973

Table S4. The ratio of I_D/I_G in Raman results of Pt@NMC, NMC and commercial Pt/C. Related to Figure 2.

Samples	I_D/I_G
1Pt@NMC	0.91
2Pt@NMC	0.93
3Pt@NMC	0.99
5Pt@NMC	0.96
NMC	0.98

Table S5. Specific surface area and pore volume results of Pt@NMC, NMC and commercial Pt/C. Related to Figure 2.

Samples	Specific Surface Area (m^2/g)	Pore Volume (cm^3/g)	Micropores Volume (cm^3/g)	Mesopores Volume (cm^3/g)
1Pt@NMC	640.8	1.205	0.09	1.115
2Pt@NMC	601.2	1.151	0.083	1.068
3Pt@NMC	516.3	0.941	0.098	0.843
5Pt@NMC	597.6	1.139	0.108	1.031
Pt/C	151.6	0.22	0.044	0.176
NMC	864.3	1.25	0.17	1.08

Table S6. The basic information summary of Pt@NMC and the control samples. Related to Figure 2.

Samples	Pt_wt(wt%)	Surface_area (m^2 g^{-1})	N_doped (wt%)
1Pt@NMC	4.6	640.8	6.3
2Pt@NMC	7.7	601.2	10.0
3Pt@NMC	13.3	516.3	10.4
5Pt@NMC	21.0	597.6	5.7
Control-1	11.6	488.9	8.2
Control-2	8.5	354.2	5.8

Table S7. The relative error of experimental results and the prediction of ML. Related to Figure 4.

		pH=14			pH=0.3		
**Sample**	**Current-Density (mA cm^{-2})**	**η_{Exp} (mV)**	**η_{Pred} (mV)**	**Relative error (%)**	**η_{Exp} (mV)**	**η_{Pred} (mV)**	**Relative error (%)**
Commercial Pt/C	10	28.9	54.9	89.8	36.3	42.2	16.3
	20	53.6	98.4	83.6	45	56.5	25.5
	40	97.6	132.8	36.1	57.7	67.2	16.5
	50	118.3	136.7	15.6	66.3	75.7	14.2
	100	217.6	186.8	14.1	103	100.8	2.1
1Pt@NMC	10	58.3	47.6	18.3	43	41.3	3.9
	20	98.9	89.4	9.6	57	58.4	2.5
	40	160.3	117.7	26.6	82.3	64.3	21.9
	50	188.9	134.9	28.6	94.3	66.6	29.4
	100	312.3	180.9	42.1	155.7	78.6	49.5
2Pt@NMC	10	21.6	47.6	120.4	38	29.8	21.6
	20	46.9	87.6	86.8	52.3	44.2	15.4
	40	84.9	109.7	29.2	76.3	68.0	10.9
	50	112.9	129.5	14.7	91	71.2	21.8
	100	211.6	179.1	15.4	151	89.0	41.1
3Pt@NMC	10	21.6	44.0	103.8	35.7	34.3	3.9
	20	46.3	93.7	102.3	48.3	55.4	14.7
	40	87.6	140.2	60.0	69	70.8	2.6
	50	106.9	165.7	55.0	79.7	72.6	8.9
	100	190.3	204.1	7.3	131	88.2	32.6
5Pt@NMC	10	23.6	40.8	73.0	39	42.1	7.9
	20	44.3	93.1	110.1	51.7	70.0	35.4
	40	83.6	131.2	56.9	73.7	81.7	10.8
	50	102.3	153.8	50.3	85	82.7	2.7
	100	183.6	179.3	2.4	139	101.7	26.8
